# Transferosome Delivery Unlocks the Therapeutic Potential of Imiquimod for Experimental Cutaneous Leishmaniasis

**DOI:** 10.3390/microorganisms14102377

**Published:** 2026-10-16

**Authors:** Gabriela V. Araujo Flores, Márcia D. Laurenti, Brayan J. Anaya, Italo Novais Cavallone, Aikaterini Lalatsa, Sarah Santos de Lima Melchert, Thaise Y. Tomokane, Sergio A. Sánchez, Dolores R. Serrano, Luiz Felipe D. Passero

**Affiliations:** 1 Institute of Biosciences, São Paulo State University (UNESP), Praça Infante Dom Henrique, s/n, São Vicente 11330-900, Brazil; gabyaraflo@gmail.com (G.V.A.F.); sarahkslima@gmail.com (S.S.d.L.M.); 2 Institute for Advanced Studies of Ocean (IEAMAR), São Paulo State University (UNESP), Rua João Francisco Bensdorp, 1178, São Vicente 11350-011, Brazil; 3 Laboratory of Pathology of Infectious Diseases (LIM50), Department of Pathology, Medical School, São Paulo University, São Paulo 01246-903, Brazil; mdlauren@usp.br (M.D.L.); thaisey@usp.br (T.Y.T.); 4 Department of Pharmaceutics and Food Technology, Faculty of Pharmacy, Complutense University of Madrid, Plaza Ramon y Cajal s/n, 28040 Madrid, Spain; branaya@ucm.es (B.J.A.); sergsa16@ucm.es (S.A.S.); dr.serrano@farm.ucm.es (D.R.S.); 5 CRUK Formulation Unit, School of Pharmacy and Biomedical Sciences, University of Strathclyde, John Arbuthnot Building, Robertson Wing, 161 Cathedral St, Glasgow G4 0RE, UK; aikaterini.lalatsa@strath.ac.uk; 6 Instituto Universitario de Farmacia Industrial, Universidad Complutense de Madrid, 28040 Madrid, Spain

**Keywords:** cutaneous leishmaniasis, *Leishmania* (*Leishmania*) *amazonensis*, therapy, imiquimod, transferosomes

## Abstract

Repurposing existing drugs offers an attractive strategy for neglected infectious diseases, but therapeutic efficacy may be limited by inadequate drug delivery to the site of infection. Imiquimod (IQ), a topical immunomodulatory agent, has shown potential against cutaneous leishmaniasis (CL); however, strategies to enhance its local therapeutic effect while minimizing drug exposure remain limited. Here, we developed IQ-loaded transferosomes (IQ-TFs) as nano-enabled topical delivery systems and evaluated their pharmaceutical performance, skin delivery properties, and antileishmanial efficacy against experimental CL caused by *Leishmania* (*L*.) *amazonensis*. IQ-TFs, prepared by thin-film hydration, showed a size of 596 ± 73 nm, low polydispersity (0.20 ± 0.02), zeta potential (−15.2 ± 1.8 mV), and high encapsulation efficiency (71.2 ± 18.7%). Encapsulation in transferosomes promoted sustained permeation and prolonged cutaneous retention of IQ, consistent with localized drug delivery. IQ-TFs markedly enhanced antileishmanial activity against both promastigotes and intracellular amastigotes. In vivo, topical IQ-TF significantly reduced parasite burden and improved infection-associated histopathological alterations. Notably, IQ-TFs achieved these effects using approximately 30-fold less IQ than the commercial 5% cream, with parasite control comparable to systemic deoxycholate amphotericin B. These findings highlight IQ transferosomes as a promising nanotechnology platform for transforming imiquimod into a more efficient low-dose topical therapy for cutaneous leishmaniasis.

## 1. Introduction

Leishmaniasis is a parasitic disease of worldwide significance, caused by protozoan parasites that belong to the *Leishmania* genus, which comprises several species and is transmitted to humans and other mammals through the bite of infected female phlebotomine sandflies, belonging to the genus *Lutzomyia* in the Americas [1,2]. Clinically, leishmaniasis is classified into cutaneous and visceral forms, the latter being potentially fatal if left untreated. Disease manifestation depends on multiple factors, including the infecting *Leishmania* species, the host immune response, and the vector involved [3].

Localized cutaneous leishmaniasis is the most prevalent clinical form and is caused by dermotropic species of the *Viannia* and *Leishmania* subgenera. *Leishmania* (*L*.) *amazonensis* is of particular relevance in the Americas due to the broad spectrum of clinical manifestations it produces. This species is mainly associated with localized cutaneous leishmaniasis and anergic diffuse cutaneous leishmaniasis. Infection with *L*. (*L*.) *amazonensis* can result in ulcerated skin lesions and, in some cases, can progress to disseminated disease when the host immune response fails to control parasite replication, leading to severe complications and permanent disfigurement [3,4]. These clinical manifestations, together with the limitations of currently available therapies, highlight the need for safer and more effective treatment strategies.

Currently, only a limited number of therapeutic options are available for the treatment of human leishmaniasis, and their efficacy varies according to the *Leishmania* species and clinical form of the disease. Pentavalent antimonials have been the mainstay of treatment for decades; however, their clinical use is limited by considerable toxicity. The most common adverse effects include anorexia, vomiting, nausea, abdominal pain, malaise, myalgia, arthralgia, headache, metallic taste, and lethargy. Hepatotoxicity and cardiotoxicity have also been reported, the latter being dose- and treatment duration-dependent, with T-wave inversion, QT interval prolongation, and arrhythmias among the most frequent cardiac manifestations [5,6,7]. Moreover, the emergence of drug-resistant *Leishmania* parasites, particularly *L*. (*L*.) *donovani*, has become an increasing challenge, further limiting the effectiveness of currently available therapies [8,9]. Consequently, second-line drugs such as pentamidine, miltefosine, paromomycin, and amphotericin B (AmB) are often required in cases of treatment failure. However, these alternatives are also associated with important limitations, including toxicity, high cost, and, in most cases, the need for parenteral administration [9,10,11].

Amphotericin B, a polyene antibiotic produced by *Streptomyces nodosus*, is considered a reference drug for leishmaniasis treatment, particularly in regions with high rates of antimonial resistance [12,13]. The antileishmanial activity of this drug is attributed to its high affinity for ergosterol in the parasite membrane, leading to pore formation, membrane destabilization, and parasite death [14,15]. However, the clinical use of AmB is limited by severe adverse effects, especially nephrotoxicity, which has driven the development of alternative drug delivery strategies aimed at reducing its toxicity while maintaining efficacy [14,16].

In this context, nano-enabled drug delivery systems have emerged as promising tools to improve therapeutic outcomes in cutaneous leishmaniasis. Among these, transferosomes, ultradeformable lipid vesicles that combine features of liposomes and elastic vesicles, have gained attention due to their ability to cross the stratum corneum and reach deeper skin layers, enabling localized and targeted drug delivery with reduced systemic toxicity [17,18]. Previous studies demonstrated that AmB-loaded transferosomes effectively enable permeation of AmB across the skin, achieving therapeutic concentrations in the epidermis and promoting potent in vivo leishmanicidal activity without detectable toxicity [12]. These findings highlight transferosomes as a versatile platform for topical drug delivery in cutaneous leishmaniasis, especially for entrapping drugs with low solubility.

Beyond conventional antiparasitic drugs, immunomodulatory compounds have been explored as alternative therapeutic strategies or as part of combination therapies. Imiquimod (IQ) is an immune response modifier approved for topical use as a 5% cream formulation (Aldara^®^, 3M Pharmaceuticals) and has been widely used in dermatological diseases, including genital warts, actinic keratoses, chromoblastomycosis, and superficial basal cell carcinoma [19,20,21,22]. The imiquimod mechanism of action involves activation of Toll-like receptor 7 (TLR7), leading to the induction of proinflammatory cytokines, including interferon-α (IFN-α), tumor necrosis factor-α (TNF-α), and interleukins, which are important inflammatory mediators to eliminate intracellular pathogens, such as *Leishmania* amastigote forms [22,23].

Preclinical and clinical studies have demonstrated that imiquimod can reduce parasite burden and accelerate lesion healing in cutaneous leishmaniasis, either alone or as an adjuvant to conventional therapies [24,25,26,27,28]. These findings support the potential of imiquimod as a topical immunotherapeutic agent, particularly when combined with advanced drug delivery systems that enhance its penetration and activity at the site of infection.

One of the main challenges in leishmaniasis treatment is the intracellular localization of *Leishmania* amastigotes within macrophage phagolysosomes, which limits drug access to the target site [29]. Lipid-based nanocarriers, such as transferosomes, offer advantages in this regard due to their biocompatibility, enhanced skin permeation, and improved macrophage uptake, potentially increasing drug concentration at the site of infection, while minimizing adverse effects [30,31].

Given the current limitations of therapies and the necessity for safer and more effective topical treatments, this study seeks to assess the efficacy of imiquimod loaded in transferosomes against experimental cutaneous leishmaniasis using a strain of *L*. (*L*.) *amazonensis*, a species associated with anergic diffuse cutaneous leishmaniasis in humans in the New World. Additionally, the study compared the activity of imiquimod-loaded transferosomes with that of the reference drug, amphotericin B, to assess imiquimod’s potential as a treatment for cutaneous leishmaniasis.

## 2. Materials and Methods

### 2.1. Materials

Imiquimod (IQ) (99.5%) and amphotericin B (≥98%) (AmB) were obtained from Ningbo Vitax Biotech (Ningbo, China) and North China Pharmaceutical Huasheng Co. Ltd (Shijiazhuang, China), respectively. Commercial cream containing 5% imiquimod (Modik^®^, EMS S/A, São Paulo, Brazil) was commercially purchased in São Paulo, Brazil. AmB was used as a positive control and as standard treatment throughout the manuscript.

Phosphatidylcholine (Lipoid S100) was kindly donated by Lipoid GmbH (Ludwigshafen, Germany). Sodium deoxycholate (NaDC) was purchased from Sigma-Aldrich (Madrid, Spain). Unless otherwise specified, all other reagents were of ACS grade (Sigma-Aldrich, Madrid, Spain) and were used without further purification. High-performance liquid chromatography (HPLC)-grade solvents were obtained from Fisher Scientific (Madrid, Spain).

### 2.2. Preparation of Imiquimod-Loaded Transferosomes

Transferosomes containing IQ (IQ-TF) were prepared using the thin-film hydration method as described by Fernández-García et al., 2020 [12]. Briefly, IQ (7.28 mg), S100 Lipoid phosphatidylcholine (686.96 mg), and NaDC (113.04 mg) were dissolved in methanol. Blank transferosomes (Blank-TFs) were prepared following the same procedure, except that IQ was omitted from the formulation. The organic solvents were evaporated under vacuum and protected from light using a rotary evaporator RV 06-ML (Janke & Kunkel Ika-Labortechnik, Staufen, Germany) at 43 ± 2 °C, rotating at 250 rpm and connected to a Vac V-500 vacuum pump (Büchi Labortechnik, Flawil, Switzerland), and controlled by a B-721 vacuum controller (Büchi Labortechnik, Flawil, Switzerland) until a dry lipid film was formed. The resulting film was hydrated with 1 × PBS (0.01 M phosphate-buffered saline, pH 7.4) and ethanol (91.5:8.5, *v*/*v*) for 30 min under continuous rotation, while being protected from light.

### 2.3. High-Performance Liquid Chromatography (HPLC) Quantification of IQ

The loading of IQ in transferosomes (IQ-TF) was quantified by high-performance liquid chromatography (HPLC). Peak integration was performed using Chromeleon software version 7.3.1.6535. A reverse-phase C18 Kinetex EVO 5 µm column (250 mm × 4.6 mm) was used. Isocratic elution was performed with a mobile phase composed of acetonitrile: acetate buffer 0.05 M pH 4: triethylamine (30:69.85:0.15 *v*/*v*). The flow rate was set to 0.8 mL/min, and the injection volume was 50 µL. Detection was carried out at 242 nm, and a linear calibration curve was obtained in the range of 0.03 to 31.25 µg/mL (y = 7.36x + 15.347, R^2^ = 0.983) with a retention time of 16 min. Transferosomes were diluted 1:10 (*v*/*v*) with mobile phase to obtain samples with IQ concentrations within the linear range of the calibration curve.

### 2.4. Characterization of Transferosomes

The particle size, polydispersity index (PDI), and zeta potential of freshly prepared transferosomes were determined at 25 °C using a Zetasizer Lab (Malvern Panalytical Ltd., Malvern, UK). Particle size and PDI were measured by dynamic light scattering (DLS), whereas zeta potential was determined by electrophoretic light scattering (ELS) using mixed-mode phase analysis light scattering (M3-PALS). Before analysis, samples were diluted 1:1000 (*v*/*v*) with deionized water. Measurements were performed in triplicate, with each triplicate consisting of 11 runs.

### 2.5. Preparation of IQ-TF Topical Nano-Enabled Gels

Topical gels containing either IQ-TF or Blank-TF were prepared using carbomer as the gelling agent. Carbopol 940 (1 g) was dispersed in 25 mL of deionized water under continuous stirring and was allowed to swell for 24 h. Transferosomes (1 part) were gradually incorporated into the hydrated Carbopol gel (3 parts) by dropwise addition and gently mixed using a mortar and pestle until homogeneous gels (IQ-TF: Carbopol 4% 1:3 *w*/*w*) were obtained. The pH was adjusted to 6.5 ± 0.1 using approximately 0.8 mL of sodium hydroxide (2 M), as measured with a Mettler Toledo MP 220 pH meter (Mettler Toledo, Greifensee, Switzerland). Stirring was maintained until a uniform, stable gel was achieved. Gels were used for both the in vitro permeability studies and in vivo treatment experiments. The formulations were stored at 2–8 °C until used.

### 2.6. Morphological Analysis

IQ-TF gels were diluted 1:1000 (*v*/*v*) with deionized water and their morphology was examined using transmission electron microscopy (TEM). A sample of each diluted gel (10 μL) was placed on a Formvar/carbon-coated copper grid (F196/100, 3.05 mm, 300 mesh; TAAB Laboratories, Aldermaston, UK) and excess sample was removed with Whatman no 1 filter paper, and the grids were allowed to air-dry at room temperature without negative staining. Images were acquired using a JEOL JEM-2100HT transmission electron microscope (Tokyo, Japan) operating at an accelerating voltage of 200 kV. Digital micrographs were recorded using an ORIUS SC1000 CCD camera (Gatan Inc., Pleasanton, CA, USA).

### 2.7. Rheological Characterization of Transferosome Gels

The rheological properties of the transferosome gels (1 g) were characterized using an AR 2000 rotational rheometer (TA Instruments, Elstree, UK) equipped with a 4 cm cone-and-plate geometry (Carri-Med, Elstree, UK). Measurements were performed at 32 ± 1 °C after a 2 min equilibration period, with a 1000 µm gap maintained between the cone and plate. The shear rate was increased and then decreased over a range of 0.1–100 s^−1^ to obtain the flow rheograms of each formulation. The total analysis time was 20 min. Data were acquired in logarithmic mode, with 10 points recorded per decade.

### 2.8. Adhesiveness Analysis of Gel Formulations

The adhesive properties of the gel formulations were determined using a Texture Analyzer (TA Stable Micro Systems Ltd., Godalming, UK) equipped with a 25 mm Perspex cylindrical probe (P/25P). A porcine ear skin membrane hydrated in 1× PBS was placed on the texture analyzer platform. Approximately 1 g of each gel formulation was then evenly spread over the membrane surface. The samples were subjected to a compression test at a speed of 0.5 mm/s. A trigger force of 5 g was first applied, followed by a target force of 20 g, which was maintained for 10 s. The probe was subsequently withdrawn over a return distance of 10 mm. Force–distance curves were recorded throughout the compression–withdrawal cycle to characterize the adhesive behavior of the gel formulations.

### 2.9. Transcutaneous Permeability In Vitro Study

Porcine ear skin was obtained from 3-month-old male pigs at a slaughterhouse. The skin from these ears was used to analyze the permeation profile of IQ-TF. Briefly, the freshly excised ears were shaved, and the underlying muscle tissue and hypodermis were manually removed by immersing the ears in 1× PBS for 30 s. The skin membranes were prepared from the ears with a thickness of 1.39 ± 0.06 mm and stored flat in plastic bags at −20 °C until further use within 1 month. Skin membranes was thawed in 1× PBS preheated to 37 °C 30 min prior to the onset of permeability studies. The skin was mounted between the donor and receptor compartments of Franz diffusion cells, with a diffusion area of 1.766 cm^2^. The receptor compartment was filled with 12 mL of 1× PBS. The donor compartment was prefilled with 1× PBS for 30 min at 32 °C. After this period, the PBS was removed, and 1 g of IQ-TF gel was applied to the donor compartment in contact with the membrane. Samples (1 mL) were withdrawn from the receptor compartment at predetermined time points (15, 30, 45, 60, 90, 120, 180, 240, 300, and 360 min) and immediately replaced with an equal volume of fresh receptor medium pre-equilibrated at 37 °C to maintain sink conditions and a constant receptor volume. All samples were stored at −20 °C until HPLC analysis.

At the end of the permeation experiment (360 min), the skin surface was gently cleaned with a cotton swab moistened with 1× PBS to remove any residual formulation. The stratum corneum was then sequentially removed using ten adhesive tape strips (13 mm diameter, Scotch^®^, 3M Company, St. Paul, MN, USA). Each tape was placed on the membrane surface, subjected to a weight of 100 g for 2 s [12], and then carefully removed. The collected tape strips were individually transferred to Eppendorf tubes containing 1 mL of the mobile phase, vortex-mixed for 1 min, and centrifuged at 1733× *g* for 10 min. The retained imiquimod was subsequently quantified by HPLC as described previously.

Regression analysis was used to calculate the slopes and intercepts of the linear portion of each graph. The steady-state flux (J_SS_), permeability coefficient (P), the diffusion coefficient, and the lag time were estimated as previously described by Serrano et al. [32]. The formulation was tested at least in triplicate.

### 2.10. Ethical Statement

All animal procedures were approved by the Committee of Ethics in the Use of Animals and the Brazilian National Council for the Control of Animal Experimentation of the Faculty of Medicine, University of São Paulo (protocol #1963/2023, 13 July 2023), and were conducted in accordance with national guidelines for the care and use of laboratory animals. Animals were obtained from the University of São Paulo Medical School Bioterism Center, housed under standard conditions with food and water ad libitum, and anesthetized with ketamine and xylazine before euthanasia with sodium thiopental.

### 2.11. Parasites

The parasite *L*. (*L*.) *amazonensis* (MHOM/BR/1973/M2269) was identified using monoclonal antibodies and isoenzyme electrophoretic profiles at the Leishmaniasis Laboratory of the Evandro Chagas Institute (Belém, Pará State, Brazil). *L*. (*L*.) *amazonensis* was grown in Schneider’s Drosophila medium (Sigma-Aldrich Co., St. Louis, MO, USA), supplemented with 10% heat-inactivated fetal bovine serum (Gibco^®^; Thermo Fisher Scientific, Waltham, MA, USA), 10 μg/mL gentamicin, and 100 U/mL penicillin (Thermo Fisher Scientific, Waltham, MA, USA) (S10 medium) at 25 °C. Stationary growth-phase promastigote forms were used in all experiments.

### 2.12. Bone Marrow Macrophages (BMMs)

Seven-week-old BALB/c mice were euthanized in a CO_2_ chamber; asepsis was performed with 70% alcohol, the femur and tibia were collected in a laminar flow, and bone marrow was removed from these bones using Hanks’ balanced salt solution (HBSS). The pellet obtained after centrifugation was incubated with 10 mL of lysis solution (0.17 M NH4Cl, pH 7.4) for 2 min at 4 °C to lyse the erythrocytes, then the reaction was stopped by adding 35 mL of HBSS. The cells were centrifuged at 180× *g* for 5 min at 4 °C, the supernatant was discarded, and the cells were cultured in Petri dishes containing RPMI 1640 medium supplemented with 10% fetal bovine serum, 2 mM L-glutamine, 10 mM Hepes, 1 mM sodium pyruvate, 1% *v*/*v* non-essential amino acid solution, 10 µg/mL gentamicin and 100 U/mL penicillin (R10 medium), as well as 15% (*v*/*v*) L929 cell-conditioned medium (LCM) as a source of macrophages colony-stimulating factor-1 (CSF-1). Cells were incubated at 37 °C with 5% CO_2_. After 24 h, the nonadherent cells were collected and cultured for one week in sterile Petri dishes with R10 medium containing 15% LCM. On days 2, 3, 5, and 7, fifteen percent LCM was added to the culture. On day 8, adherent cells were carefully collected, counted, and plated at a concentration of 10^6^ macrophages/well in sterile 96-well plates or on round glass coverslips, depending on the experiment.

### 2.13. In Vitro Evaluation of Leishmanicidal Potential and Cytotoxicity

In vitro tests were conducted to evaluate the efficacy of the formulations and drugs on the promastigote forms of *L*. (*L*.) *amazonensis*. Promastigote forms were cultured and collected by centrifugation (1889× *g*, 10 min, 4 °C) and plated in 96-well plates at a concentration of 2 × 10^6^ promastigotes/well in S10 medium. Serial dilutions of IQ-TF and Blank-TF (2 to 1000 ng/mL), IQ (156 to 20,000 ng/mL), and AmB (3.9 to 5,000 ng/mL) were added to the parasites and incubated at 25 °C. After 24 and 72 h, the parasites were centrifuged at 1889× *g*, 4 °C for 10 min, and the supernatant was discarded. The parasites were washed three times with 200 µL of sterile 1× PBS to ensure drug removal. A cell viability assay based on resazurin oxidation (Thermo Fisher Scientific, Waltham, MA, USA) was performed using a final resazurin concentration of 44 µM. This was achieved by diluting the 100× resazurin solution 1:10 in RPMI medium (one-part resazurin solution and nine parts RPMI medium). After 1 h at 25 °C, fluorescence was measured in a spectrophotometer, with excitation at 535 nm and emission at 615 nm (GloMax^®^-Multi+ Detection System, Promega^©^, Madison, WI, USA). The efficacy of the compounds was expressed as the concentration that eliminated 50% of the parasite population (Effective Concentration 50%—EC_50_), determined from dose–response curves using the GraphPad Prism 10 program.

The cytotoxic effects of the drugs listed in the item drugs were evaluated in BMM (10^6^ cells/well) cultured in 96-well plates in R10 medium together with serial dilutions of IQ-TF, Blank-TF (2 to 1,000 ng/mL), IQ (99.8 to 100,000 ng/mL), and AmB (99.8 to 100,000 ng/mL) during 24 and 72 h. During these periods, cells were maintained in an incubator at 37 °C, 5% CO_2_. At these time points, cells were centrifuged at 180× *g* for 10 min, 4 °C, and washed three times with 200 µL of sterile 1× PBS to ensure drug removal. The cell viability assay was determined as described above. The cytotoxic concentration that eliminates 50% of the cell population (CC_50_) was determined from dose–response curves using the GraphPad Prism 10 program.

### 2.14. Evaluation of Drug Potential in Parasite–Host Cell Interactions

To evaluate the anti-amastigote potential of IQ, IQ-TF, Blank-TF, and AmB, BMM from BALB/c mice (10^6^ cells/mL) were plated on sterile round coverslips and placed in 24-well plates for 24 h in a humidified incubator with 5% CO_2_ at 37 °C. After this period, the cells were infected with promastigote forms of *L*. (*L*.) *amazonensis* in stationary growth phase at a ratio of 10 parasites per macrophage and incubated at 35 °C. After 24 h, the wells were washed three times with warm sterile 1× PBS to remove non-internalized parasites, and different concentrations of IQ-TF, Blank-TF, IQ, or AmB (15 to 125 ng/mL) were added to the infected cells and incubated for 24 and 72 h. Infected controls were incubated with R10 medium only. After 24 and 72 h of incubation, coverslips were washed in sterile 1× PBS, fixed in methanol, and stained with Giemsa. The number of parasites per macrophage and intracellular amastigotes was quantified to determine the infection index, as reported by Yamamoto et al. (2015) [33]. The selectivity indices (SIs) of the tested drugs were estimated by the ratio between CC_50_ and EC_50_ [33].

### 2.15. Preclinical Studies with IQ and IQ-TF Formulation in Experimental Cutaneous Leishmaniasis

A total of 30 male BALB/c mice per experiment were subcutaneously infected at the base of the tail with 50 µL of *L*. (*L*.) *amazonensis* promastigotes (10^6^ parasite/animal), while five mice received only 0.9% (*w*/*v*) sodium chloride at the base of the tail (healthy group).

Thirty days post-infection (PI), the infected animals were randomly assigned to six experimental groups of five animals each. Group 1 received the commercially available 5% imiquimod cream (Modik^®^), which was used as the reference treatment for comparison with the experimental IQ-TF gels. The cream was applied topically three times per week, following the manufacturer’s recommended dosing regimen (Modik^®^, EMS S/A, São Paulo, Brazil). Group 2 received an IQ-TF gel and Group 3 received a corresponding Carbopol-based gel containing blank transferosomes (Blank-TF gel). Both formulations were applied topically once daily. Group 4 received amphotericin B deoxycholate (AmB; 1 mg/kg, standard treatment) via intraperitoneal injection (100 µL), once daily. Group 5 consisted of untreated infected animals, and Group 6 consisted of uninfected, untreated healthy animals.

The treatment regimen was maintained for 10 days. For the topical administration groups (Groups 1–3), the formulations were standardized to an application amount of 17 mg/animal. The formulations were then applied directly to the lesion site at the base of the tail to ensure uniform coverage of the entire lesion surface without excessive overflow. Over the treatment period, this corresponded to 0.0279 mg of imiquimod per application for the IQ-TF group and 0.85 mg per application for the commercial IQ cream group. Thus, the cumulative imiquimod dose was 0.279 mg/animal for the IQ-TF group (10 daily applications) and 4.25 mg/animal for the commercial IQ cream group (5 applications administered three times per week).

### 2.16. Lesion Progression and Parasite Load

The inoculation area of the parasites in the treated groups and the infected control group was measured weekly, before, during, and after treatment, using a digital caliper (Mitutoyo Sul Americana Ltda., São Paulo, Brazil). Lesion size was determined by measuring the height and width of the tail at the lesion site. To account for the physiological thickness of the tail, baseline measurements obtained from healthy control animals were used as reference values. The lesion size was calculated as the difference between the measurements of infected animals and the corresponding baseline values, and the final lesion area was expressed in square millimeters (mm^2^).

Parasite load (PL) was determined using the limiting dilution assay [34,35]. Briefly, a fragment of the lesion was collected, debrided, and weighed, then macerated in S10 medium. A volume of 150 µL of the homogenate was added to a sterile 96-well plate in quadruplicate, and serial 1:3 dilutions were performed in twelve replicates per dilution. Plates were incubated at 25 °C for 10 days to observe promastigote forms. During this period, each plate was monitored, and the highest dilution in which promastigotes were observed was recorded. Tissue parasite load was calculated according to Lima et al. [36].

The parasite load was expressed as the number of parasites per milligram of tissue.

### 2.17. Histopathological Study

A fragment of skin was fixed in 10% formalin buffered with 0.01 M phosphate buffer for 24 h, processed using the usual histology techniques, and embedded in paraffin to obtain the sections. Histological sections stained with hematoxylin–eosin (HE). To evaluate the skin tissue lesions, a semi-quantitative comparative analysis was performed according to the modified method of Ridley and Ridley [37,38]. The results were scored based on the intensity of each tissue change observed using a 40× objective as follows: (1) discrete, (2) moderate, (3) severe and (4) extremely severe [37,38].

### 2.18. Statistical Analysis

For statistical analysis, GraphPad Prism 11 software (San Diego, CA, USA) was used. Parametric data comparing two independent groups were evaluated using an unpaired Student’s *t*-test. For comparisons among three or more groups with a single independent variable, a one-way analysis of variance (ANOVA) followed by Bonferroni’s post hoc test was applied. Data involving two independent variables were analyzed using a two-way ANOVA followed by Tukey’s post hoc test for multiple comparisons. Differences were considered statistically significant when *p* < 0.05. All experiments were independently repeated three times, and the results are expressed as the mean ± standard error.

## 3. Results

### 3.1. Physicochemical Characterization of IQ-TF

The physicochemical characterization of IQ-TF and Blank-TF is presented in Table 1. IQ-TF large unilamellar vesicles showed moderate colloidal stability (595.7 ± 73.1 nm, −15.2 ± 1.8 mV) and low polydispersity (PDI: 0.2 ± 0.02). Unloaded particles were of similar size but with a wide polydispersity. The theoretical IQ content in IQ-TF gels was 0.23% (*w*/*w*) and experimental HPLC quantification indicated an encapsulation efficiency of 71.2 ± 18.7%. Thus, a drug loading of 0.164% (*w*/*w*) was obtained, which corresponds to 1.64 mg IQ/g of the TF gel formulation.

### 3.2. Morphological Analysis of IQ-TF Gel Formulation

**Figure 1:**
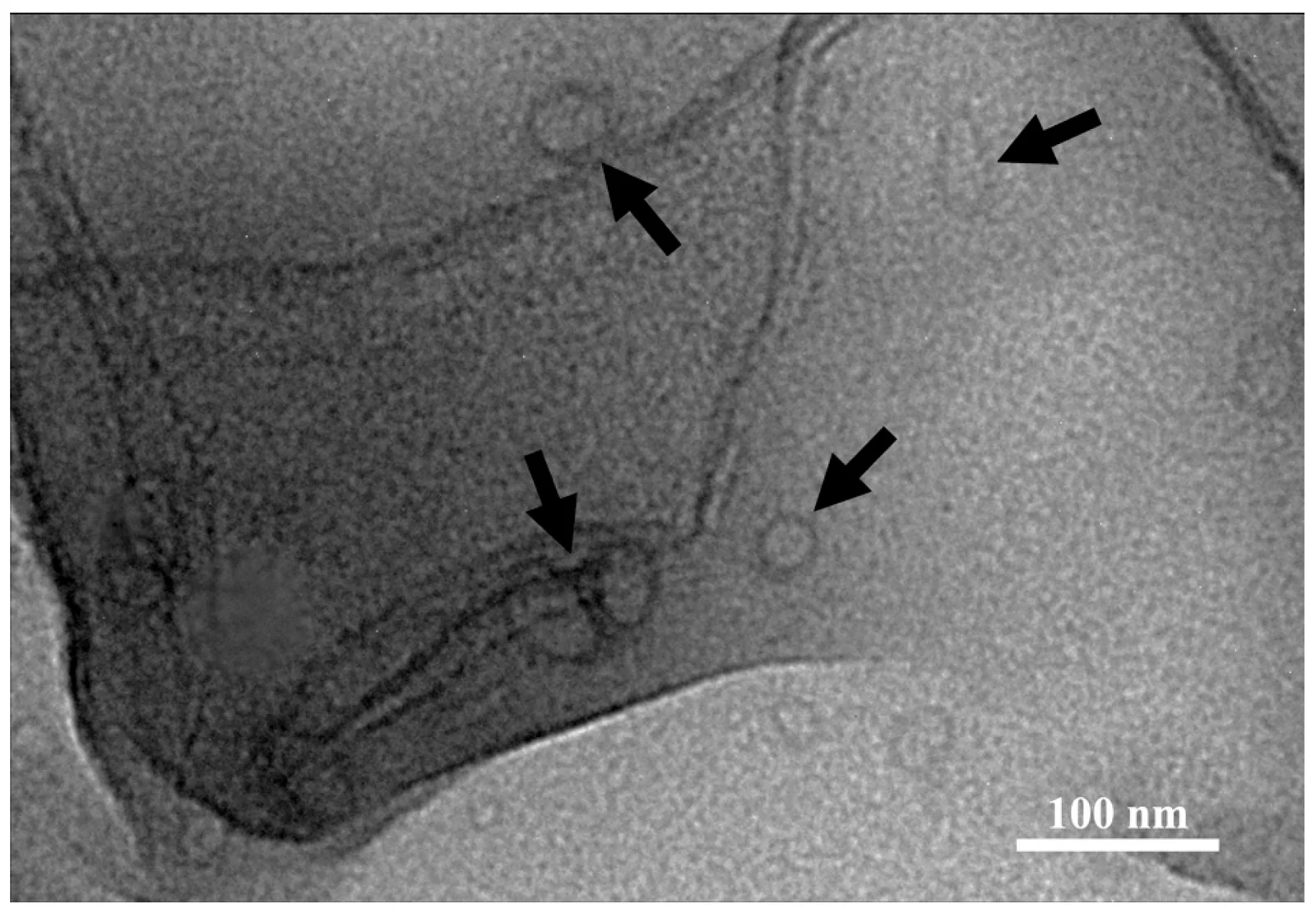
Representative unstained transmission electron microscopy (TEM) micrograph of IQ-loaded transferosome (IQ-TF) gel formulation, showing well-defined vesicular structures with predominantly spherical morphology and flexible lipid membranes. Black arrows indicate representative transferosome vesicles. Scale bar: 100 nm.

Transmission electron microscopy (TEM) images of IQ-TF revealed a well-defined spherical particle with vesicular morphology of uniform size; however, the double bilayer was not clearly visible in the absence of staining (Figure 1).

### 3.3. Rheological Characterization of Transferosome Gels

**Figure 2:**
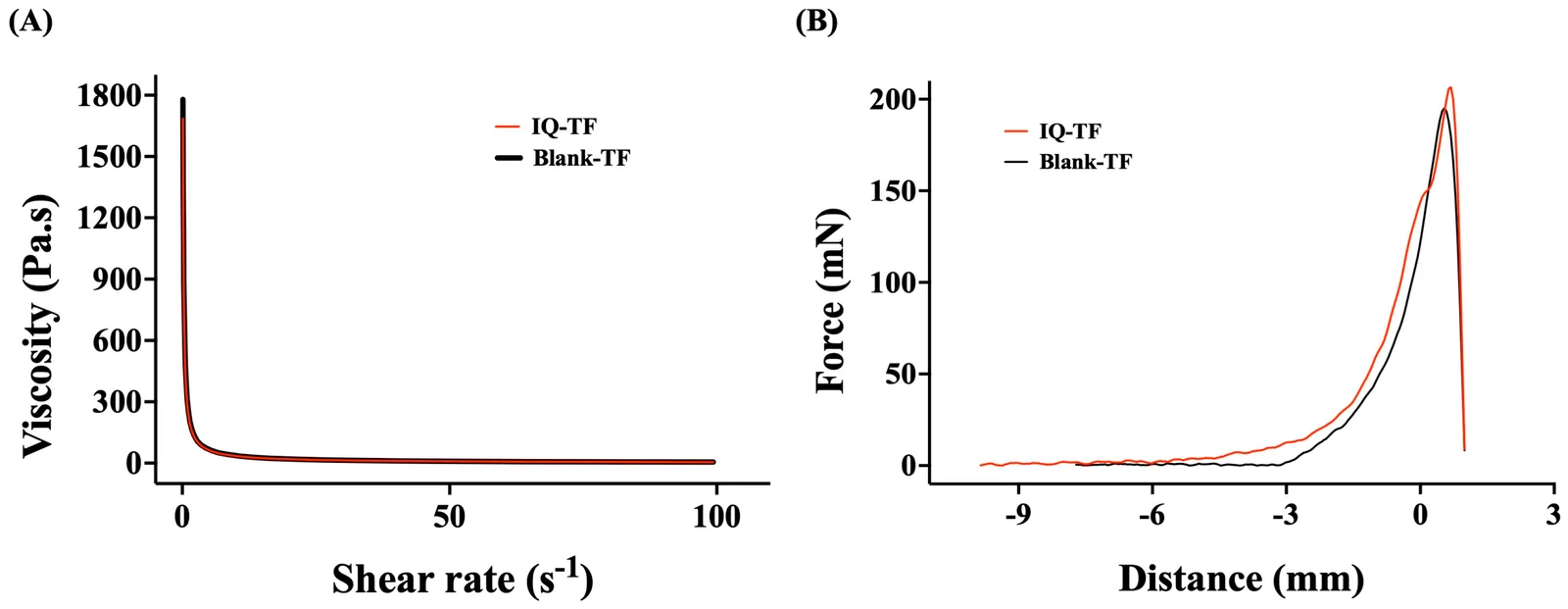
Rheological and adhesiveness profiles of IQ-loaded transferosome (IQ-TF) and blank transferosome (Blank-TF) topical gel formulations. (**A**) Rheological profiles. (**B**) Representative adhesiveness profiles. IQ-TF and Blank-TF are represented by red and black lines, respectively.

Both transferosome gels were shear thinning with increasing shear rates, confirming their non-Newtonian, pseudoplastic behavior (Figure 2A). The viscosity at a shear rate of 100 s^−1^ was 5.05 ± 3.57 Pa·s for IQ-TF and 5.06 ± 3.58 Pa·s for Blank-TF, demonstrating the two formulations’ comparable rheological properties mostly driven by the gelling agent. This is expected as mean particle size was similar, although Blank-TF had a high polydispersity index, which is likely to overall describe viscosity of the gel.

### 3.4. Adhesiveness Analysis

The texture profiles of the IQ-TF and Blank-TF topical gels are shown in Figure 2B. Both gels exhibited force–distance curves characteristic of semi-solid systems. As the probe entered the gel, there was a progressive increase in compressive force, which reached a maximum before probe withdrawal. This was followed by a sharp decrease in force during probe removal. The nearly overlapping profiles suggest that loading imiquimod into transferosomes does not significantly impact on the mechanical properties of the Carbopol gel. Overall, both formulations exhibited comparable mechanical behavior, consistency, and structural integrity.

### 3.5. In Vitro Skin Permeation

**Figure 3:**
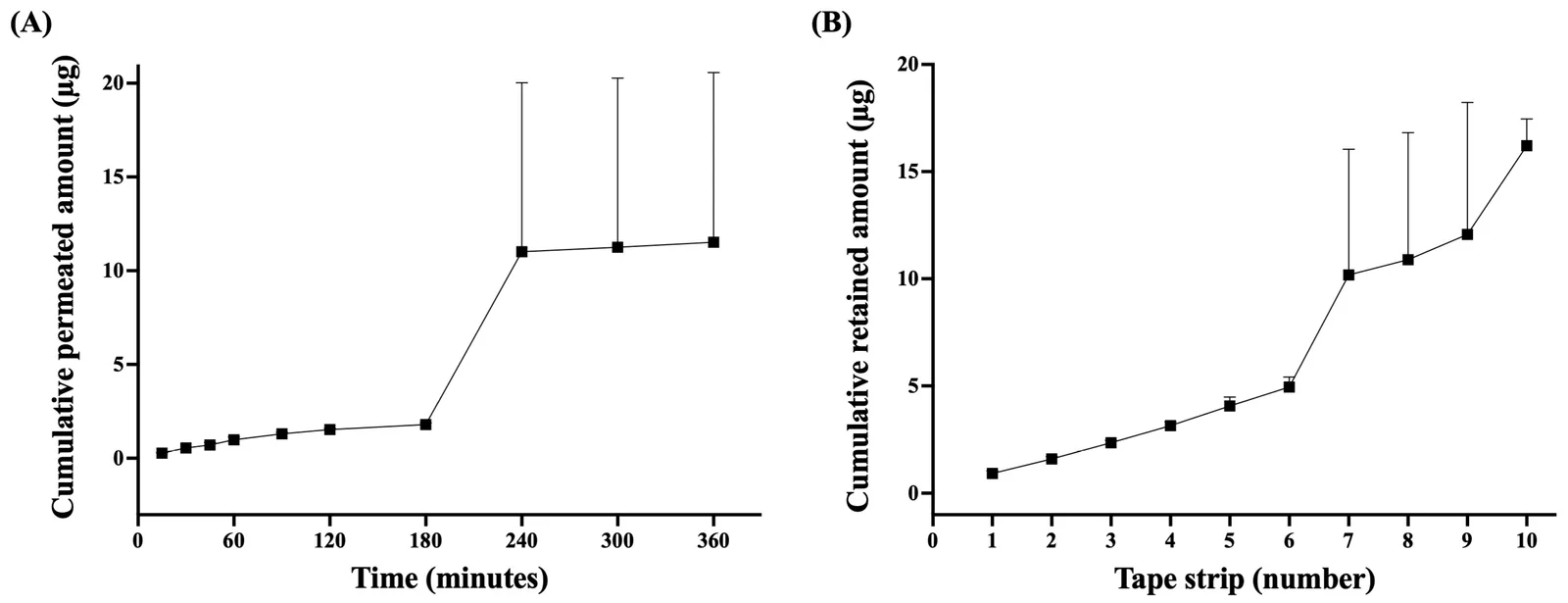
In vitro permeation and skin retention of IQ-TF across porcine ear skin. Key: (**A**)—the cumulative amount of imiquimod that permeated through the skin. (**B**)—the cumulative amount of imiquimod retained in the stratum corneum after tape stripping at the end of the permeation experiment. Skin retention was determined using ten consecutive adhesive tape strips. Data are presented as mean ± standard error.

IQ-TF exhibited a progressive increase in the cumulative amount of permeated imiquimod throughout the 6 h experiment, reaching a final cumulative amount of 11.52 ± 9.05 µg (Figure 3A and Table 2). The formulation showed a steady-state flux (J_ss_) of 0.858 ± 0.132 µg/cm^2^/h, a lag time of 0.894 ± 0.005 h, a permeability coefficient of 5 × 10^−4^ ± 1 × 10^−4^ cm^2^/h, and a diffusion coefficient of 6.8 × 10^−4^ ± 0.1 × 10^−4^ cm^2^/h (Table 2). These results demonstrate that IQ-TF enabled the continuous permeation of imiquimod across excised porcine skin under the experimental conditions evaluated.

### 3.6. Skin Localization (Tape Stripping)

The retention of imiquimod within the stratum corneum following the in vitro permeation experiment was evaluated by tape stripping using ten consecutive adhesive tapes. The cumulative retention profile is presented in Figure 3B. The cumulative amount of retained imiquimod increased throughout the sequential removal of the stratum corneum, reaching a final cumulative amount of 16.21 ± 1.24 µg after the tenth tape strip.

### 3.7. Antileishmanial and Cytotoxic Activity of IQ and IQ-TF

IQ-TF, Blank-TF, IQ, and AmB were evaluated for cytotoxic effect, antileishmanial activity on *L*. (*L*.) *amazonensis* promastigotes, on amastigote forms, and selectivity index (SI) (Table 3). Cytotoxicity assays in macrophages showed that IQ-TF exhibited CC_50_ values of 150 ± 1 ng/mL at 24 h and 270 ± 110 ng/mL at 72 h. Blank-TF showed CC_50_ values of 1190 ± 30 ng/mL and 570 ± 40 ng/mL after 24 and 72 h, respectively. IQ showed CC_50_ values of 35,490 ± 9540 ng/mL and 18,000 ± 5010 ng/mL after 24 and 72 h, respectively. AmB exhibited CC_50_ values of 2980 ± 70 ng/mL after 24 h and 1410 ± 90 ng/mL after 72 h.

The in vitro activity against *L*. (*L*.) *amazonensis* promastigotes revealed differences among the tested formulations and drugs. IQ-TF exhibited EC_50_ values of 50 ± 2 ng/mL after 24 h and 4.55 ± 0.01 ng/mL after 72 h. Blank-TF showed EC_50_ values of 100 ± 3 ng/mL and 530 ± 30 ng/mL. IQ presented EC_50_ values of 21,060 ± 1540 ng/mL and 12,560 ± 1410 ng/mL after 24 and 72 h, respectively. The AmB formulation exhibited EC_50_ values of 40 ± 1 ng/mL after 24 h and 56.3 ± 0.3 ng/mL after 72 h.

The antileishmanial activity of the formulations and drugs was further evaluated against intracellular *L*. (*L*.) *amazonensis* amastigotes after 24 and 72 h of incubation (Table 3). IQ-TF exhibited EC_50_ values of 4 ± 2 ng/mL and 1.2 ± 0.7 ng/mL after 24 and 72 h, respectively. In comparison, IQ showed EC_50_ values of 5800 ± 0.4 ng/mL at 24 h and 30 ± 10 ng/mL at 72 h, whereas AmB exhibited EC_50_ values of 3 ± 2 ng/mL and 10 ± 2 ng/mL at the respective time points. Blank-TF was inactive on intracellular amastigotes, as the EC_50_ value was above the highest concentration evaluated.

Regarding the selectivity index, both formulations and free drugs exhibited higher selectivity toward amastigote forms than promastigote forms after 24 and 72 h (Table 3).

### 3.8. Preclinical Studies with IQ and IQ-TF Gel Formulation in Experimental Cutaneous Leishmaniasis

**Figure 4:**
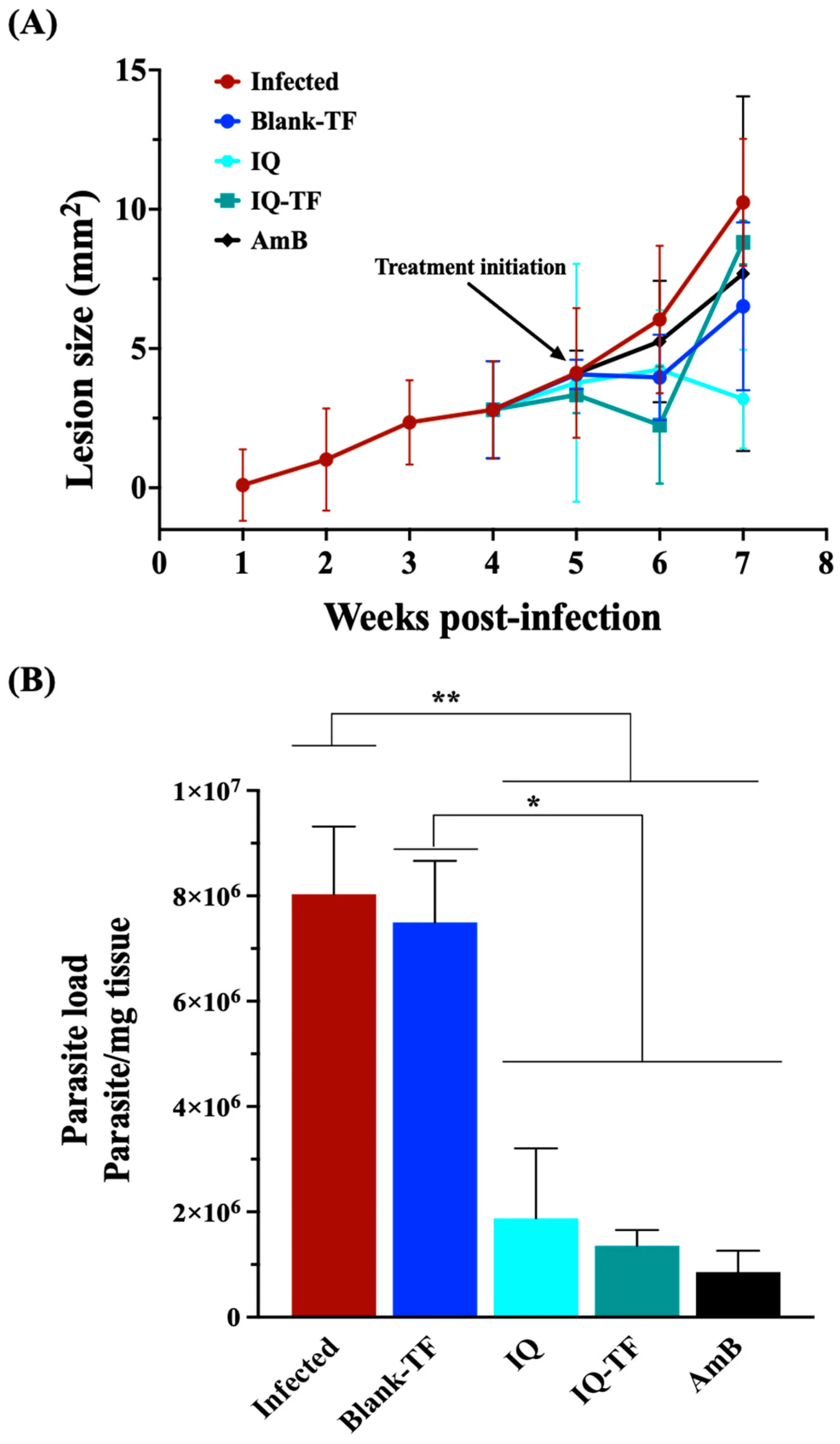
Preclinical studies of IQ-TF efficacy in experimental cutaneous leishmaniasis caused by *L*. (*L*.) *amazonensis*. (**A**) The progression of lesion size over time following treatment with commercial IQ cream (0.85 mg of IQ/application/animal), IQ-TF topical gel (0.0279 mg of IQ/application/animal), or 1 mg/kg intraperitoneal AmB, compared with untreated infected controls. Lesion size was calculated as the increase in tail area relative to baseline measurements obtained from healthy control animals and expressed in millimeters squared (mm^2^). All groups exhibited progressive increases in lesion size, with no statistically significant differences observed between treatments (*p* > 0.05). (**B**) Parasite burden was determined at the end of the experimental period by limiting dilution assay, expressed as parasites/mg tissue. Animals treated with IQ, IQ-TF, or amphotericin B showed significant reductions in parasite load compared to untreated infected controls (**: *p* = 0.01) and Blank-TF (*: *p* < 0.05). Data are expressed as mean ± standard error. Infected: untreated infected; IQ: commercial imiquimod cream; IQ-TF: imiquimod-loaded transferosomes; AmB: deoxycholate amphotericin B.

BALB/c mice infected with *L*. (*L*.) *amazonensis* were treated topically with Blank-TF gel, IQ-TF gel, commercial IQ cream (5% imiquimod cream, Modik^®^), or intraperitoneally with 1 mg/kg of deoxycholate amphotericin B as a standard treatment. Throughout the observation period, all experimental groups maintained stable body weight with no apparent signs of clinical deterioration. Furthermore, macroscopic examination of the treatment sites revealed no visible skin irritation, erythema, or other treatment-related alterations. Nevertheless, a progressive increase in lesion size was observed in all infected groups over time, regardless of treatment. Animals treated with commercial imiquimod showed a tendency toward lesion size stabilization or a slight reduction in the sixth and seventh weeks after infection compared to the control; however, no statistically significant differences were detected between groups at any evaluated time point (*p* > 0.05) (Figure 4A).

Parasite burden was determined at the end of the treatment period to evaluate the therapeutic efficacy of the different treatments against *L*. (*L*.) *amazonensis* infection (Figure 4B). As expected, the untreated infected and Blank-TF groups exhibited the highest parasite burdens (8.1 × 10^6^ ± 1.3 × 10^6^ and 7.5 × 10^6^ ± 1.2 × 10^6^ parasites/mg tissue, respectively). In contrast, animals treated with commercial IQ cream (1.9 × 10^6^ ± 1.3 × 10^6^), IQ-TF gel (1.4 × 10^6^ ± 3.0 × 10^5^), and AmB (8.6 × 10^5^ ± 4.1 × 10^5^) showed significantly reduced skin parasitism compared with the infected untreated group (*p* < 0.01) and Blank-TF group (*p* < 0.05). These values corresponded to the following reductions 76.6%, 83.1%, and 89.3%, respectively, relative to the infected untreated control. Although AmB and IQ-TF exhibited the lowest parasite burdens among the treated groups, no statistically significant differences were observed between these treatments and the commercial IQ cream group (*p* > 0.05).

### 3.9. Histopathological Study

**Figure 5:**
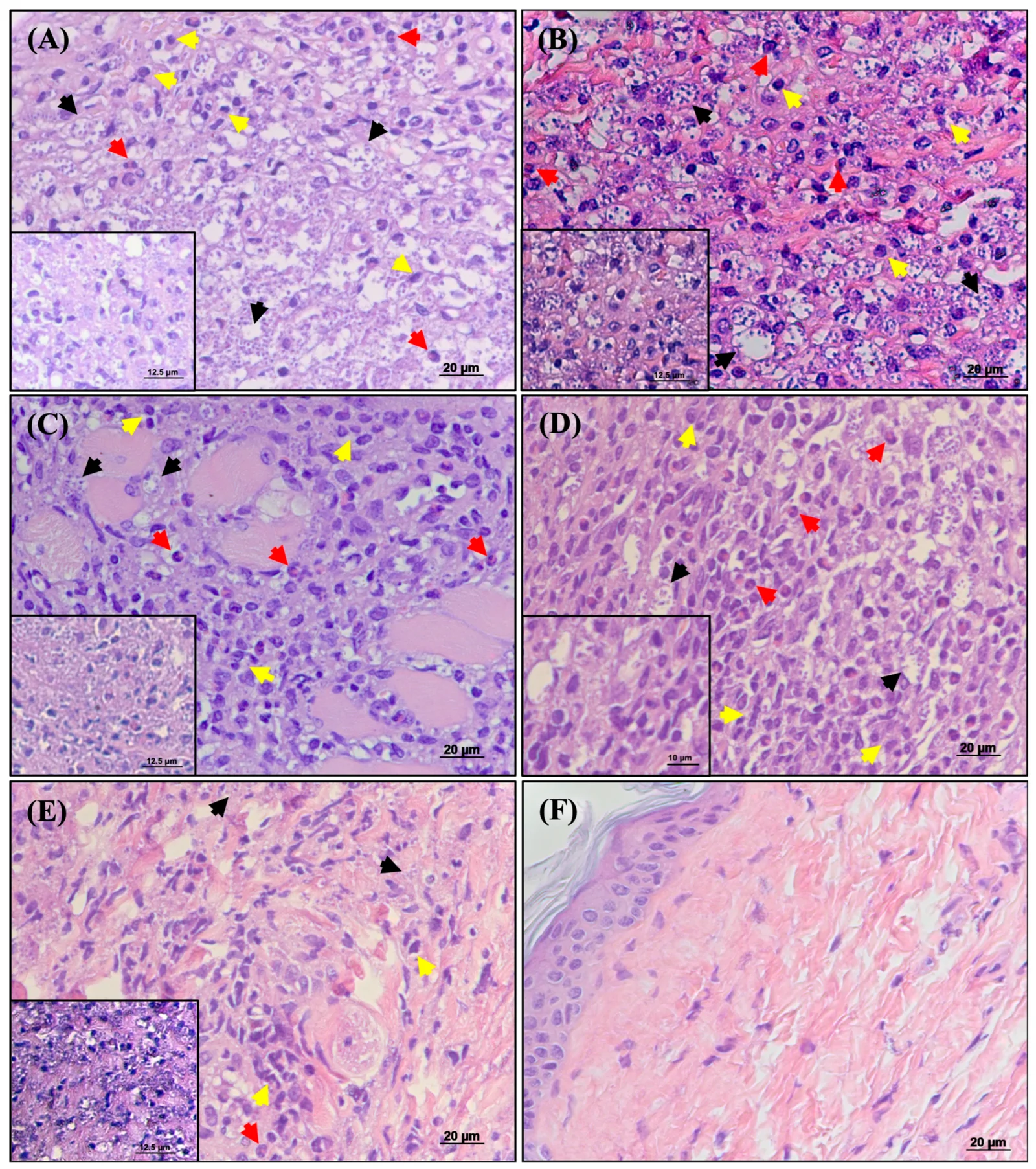
Histological skin sections of BALB/c mice infected with *L*. (*L*.) *amazonensis*. (**A**) Untreated infected control; (**B**) Blank-TF-treated control; (**C**) commercial IQ cream (17 mg/animal); (**D**) IQ-TF topical gel (17 mg/animal); (**E**) AmB (1 mg/kg, intraperitoneally, standard treatment); (**F**) uninfected untreated healthy control. Blank-TF: Carbopol-based gel containing blank transferosomes; 5% IQ cream: commercial IQ cream (Modik^®^); IQ-TF: imiquimod-loaded transferosomes; AmB: deoxycholate amphotericin B. The head arrows indicate parasitized macrophages (black), lymphocytes (red), and polymorphonuclear cells (yellow). Areas of necrosis are shown in the inset.

The histopathological examination of skin sections revealed similar inflammatory patterns with different intensity among the experimental groups (Figure 5). The infected, untreated group (Figure 5A) as well as Blank-TF-treated infected group (Figure 5B) exhibited an extremely severe, diffuse inflammatory infiltrate that extended throughout the superficial and deep dermis and into the underlying muscle. This infiltrate consisted primarily of heavily parasitized macrophages, accompanied by a few lymphocytes and polymorphonuclear cells. Focal areas of necrosis were also observed.

In contrast, animals treated with commercial IQ cream (Figure 5C) or IQ-TF gel (Figure 5D) showed differences in cellular composition of the inflammatory infiltrate and degree of macrophage parasitism compared to the untreated infected and Blank-TF groups. While a severe, diffuse inflammatory infiltrate involving both the superficial and deep dermis and into the underlying muscle persisted, the infiltrate was primarily composed of lymphocytes, followed by macrophages and polymorphonuclear cells. Unlike the infected untreated and Blank-TF groups, the macrophages exhibited moderate rather than heavy parasitism as it was observed in the controls. Polymorphonuclear cells were also more prominent in these groups than in the untreated infected and Blank-TF groups. Additionally, both groups exhibited focal areas of necrosis predominantly associated with cellular and parasitic debris.

Animals treated with deoxycholate AmB (1 mg/kg intraperitoneally, standard treatment) (Figure 5E) exhibited a severe, diffuse inflammatory infiltrate involving the superficial and deep dermis and into the underlying muscle. This infiltrate consisted of lymphocytes and macrophages in comparable proportions, together with polymorphonuclear cells. Macrophages exhibited predominantly mild to moderate rather than heavy parasitism. Unlike the IQ- and IQ-TF-treated groups, however, this group exhibited extensive areas of necrosis containing abundant cellular and parasitic debris.

In contrast, the healthy control group (Figure 5F) showed preserved epidermal and dermal architecture, with no evidence of inflammation, tissue damage, or other histopathological changes.

**Figure 6:**
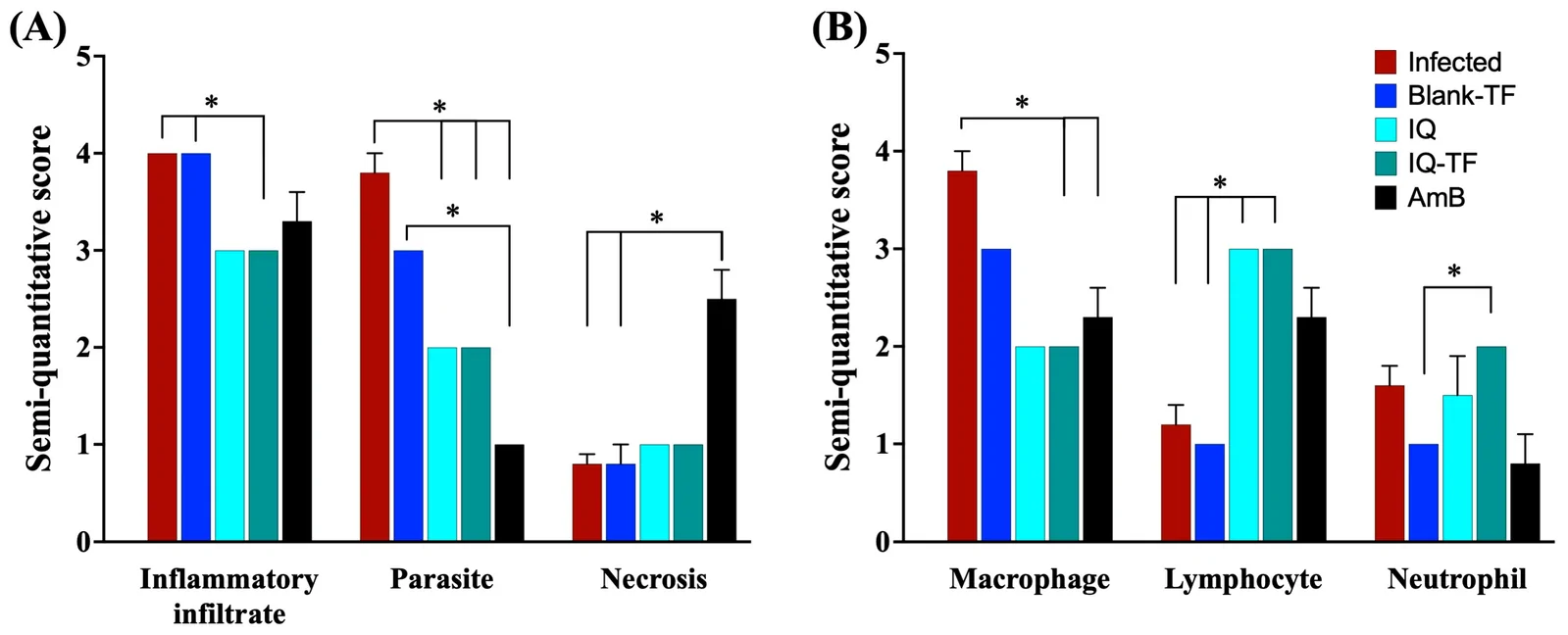
Semi-quantitative histopathological evaluation of skin lesions in BALB/c mice infected with *L*. (*L*.) *amazonensis*. Bar graphs show the mean ± standard error for (**A**) inflammatory infiltrate intensity, macrophage parasitism, and necrosis, as well as (**B**) inflammatory cell composition across the experimental groups. Histopathological features were scored on a four-point ordinal scale: (1) mild, (2) moderate, (3) severe, and (4) extremely severe, with higher scores indicating greater severity. Infected: untreated infected control; IQ: commercial imiquimod cream; IQ-TF: imiquimod-loaded transferosomes; AmB: deoxycholate amphotericin B. * *p* < 0.05.

The semi-quantitative histopathological scores for inflammatory infiltrate intensity, cellular composition, macrophage parasitism, and necrosis across the experimental groups are shown in Figure 6.

## 4. Discussion

Cutaneous leishmaniasis remains one of the most challenging neglected tropical diseases because safe, effective, and accessible therapeutic options are limited. Although several antileishmanial drugs are currently available, their clinical use is frequently compromised by prolonged treatment regimens, variable therapeutic responses, high costs, and, particularly for systemic therapies, significant adverse effects [9,39]. These limitations have driven increasing interest in alternative therapeutic strategies that improve treatment efficacy while reducing toxicity. Drug repurposing is one of these strategies that has gained increasing attention because it identifies new therapeutic applications for approved or clinically characterized compounds. This reduces the time and cost associated with conventional drug development [40,41].

Imiquimod represents one of the most promising examples of drug repurposing for cutaneous leishmaniasis [19,24,27,42]. Originally approved as a topical treatment for genital warts, actinic keratosis, and superficial basal cell carcinoma, imiquimod acts primarily as an agonist of TLR7, promoting activation of innate immune responses and triggering macrophage microbicidal activity [25,43]. Because parasite elimination in cutaneous leishmaniasis depends on the activation of infected macrophages rather than direct parasiticidal activity, imiquimod has attracted considerable attention as an immunotherapeutic agent that can enhance host defense mechanisms against *Leishmania*.

Growing evidence from experimental and clinical studies supports the therapeutic potential of imiquimod for treating cutaneous leishmaniasis. Early experimental investigations demonstrated that imiquimod activates infected macrophages, stimulates nitric oxide production, and enhances the killing of intracellular parasites, providing the biological basis for its use as an immunotherapeutic agent [25]. Subsequently, randomized clinical trials showed that combining topical imiquimod with conventional antimonial therapy significantly accelerated lesion healing, increased clinical cure rates, and improved the cosmetic appearance of residual scars compared to antimonial treatment alone [19,24,27]. On the other hand, the successful treatment of a patient with localized cutaneous leishmaniasis caused by *L*. (*L*.) *mexicana* using topical imiquimod as primary therapy has further reinforced its therapeutic potential in selected clinical settings [42]. Taken together, these findings suggest that imiquimod is a valuable treatment option and support its repositioning as a promising immunomodulatory therapy for cutaneous leishmaniasis.

Despite these encouraging findings, the therapeutic effectiveness of conventional topical imiquimod may depend on how efficiently the drug reaches infected tissues. Unlike with dermatological disorders, where the therapeutic target is primarily in the superficial epidermis, *Leishmania* amastigotes reside intracellularly within macrophages, primarily in dermal tissue. Therefore, successful topical therapy requires drug deposition not only at the skin surface, but also in the tissue compartments where the parasites reside. Consistent with this concept, Seeberger et al. evaluated topical imiquimod monotherapy and reported transient clinical improvement followed by lesion progression. This suggests that limited drug delivery to deeper skin layers may contribute to an insufficient or short-lived therapeutic response [44]. In our study, IQ-TF promoted imiquimod retention within the stratum corneum, which may be advantageous for topical treatment by favoring drug localization at the site of infection. However, stratum corneum retention does not necessarily imply delivery to deeper infected tissues. Adequate dermal distribution may also be required for sustained therapeutic activity. This consideration is particularly relevant in cutaneous leishmaniasis, in which infection-associated inflammation and tissue damage can alter the skin barrier’s integrity. Previous studies have reported negligible permeation of paromomycin sulfate through uninfected mouse skin but markedly increased permeation through *Leishmania major*-infected skin. This suggests that infection-associated alterations in skin integrity can affect drug penetration [45]. The inflammatory response and pathological changes associated with *Leishmania* infection, including ulceration and necrosis, may further disrupt the architecture of the epidermal and dermal layers. This consequently alters the skin barrier [46]. These observations suggest that the therapeutic limitations of topical imiquimod are related to its pharmacological activity and the efficiency with which it reaches its biological target in infected skin. Thus, rather than increasing the dose, strategies that optimize dermal drug delivery could enhance the therapeutic efficacy of this repurposed immunomodulator [47].

Nano-enabled medicines can not only allow improvements in drug solubility and stability but they can also significantly alter the permeability across challenging barriers such as the stratum corneum or biodistribution post-administration [44]. Thus, they are able to increase permeability across the skin, increase local drug availability, and promote sustained drug deposition within target tissues [29]. Among the various nanocarrier systems investigated for dermal drug delivery, transferosomes have attracted particular attention due to their highly deformable phospholipid membrane. This membrane allows transferosomes to penetrate the intercellular pathways of the stratum corneum more efficiently than conventional liposomes across the aqueous osmotic gradient present in the skin. This unique property makes transferosomes ideal for treating diseases such as cutaneous leishmaniasis, for which the therapeutic objective is to maximize drug delivery to infected skin, while minimizing systemic exposure [17].

Therefore, we hypothesized that incorporating imiquimod into a highly deformable transferosomes nanocarrier would improve its delivery to infected dermal tissues and maximize local therapeutic efficacy against cutaneous leishmaniasis. The first step in testing our hypothesis was to determine if the developed imiquimod transferosomes possessed the necessary physicochemical properties for efficient topical drug delivery. The incorporation of IQ did not substantially alter the size of the vesicles, as the mean particle size of IQ-TF was 596 ± 73 nm compared with 574 ± 9 nm for Blank-TF, indicating that drug loading did not compromise vesicle assembly or structural integrity, while maintaining a high encapsulation efficiency of 71.2%.

Although the mean diameter of IQ-TF was close to 600 nm, this value should be interpreted in the context of transferosome systems. Unlike conventional liposomes, transferosomes are highly deformable vesicles. Their ability to penetrate the skin depends on both particle size and the elasticity of their lipid bilayer. The presence of edge activators enables these vesicles to undergo reversible deformation, allowing them to traverse intercellular pathways considerably smaller than their own diameter [48]. In addition to particle size and deformability, the colloidal stability of the formulations is another important parameter to consider for their physicochemical characterization.

Transferosomes showed moderate colloidal stability (−15.2 ± 1.8 mV for IQ-TF and −23.6 ± 0.8 mV for Blank-TF). Transferosomes are primarily stabilized through a combination of electrostatic and steric mechanisms provided by phospholipids and nonionic surfactants, meaning that moderate zeta potential values do not necessarily indicate poor colloidal stability [49]. Moreover, colloidal stability depends on the balance between attractive van der Waals forces and electrostatic repulsion. As discussed by Bhattacharjee, when the Hamaker constant is low, the attractive van der Waals interactions become weaker, allowing even relatively low zeta potential values (approximately 10–15 mV) to provide sufficient electrostatic repulsion to maintain colloidal stability [50]. Therefore, the zeta potential values obtained for IQ-TF and Blank-TF are consistent with a stable colloidal system and suggest that the slight reduction in surface charge following imiquimod incorporation is more likely to reflect changes in lipid bilayer organization than a loss of formulation stability.

The rheological and adhesive properties of the developed gels are relevant to their topical application in the treatment of cutaneous leishmaniasis. Both IQ-TF and Blank-TF exhibited pronounced shear-thinning behavior, with viscosity decreasing from 1686.0 to 5.1 Pa·s for IQ-TF and from 1780.0 to 5.4 Pa·s for Blank-TF as the shear rate increased from 0.12 to 99.34 s^−1^. This behavior may facilitate spreading during application, while maintaining sufficient consistency under low-shear conditions, as commonly reported for Carbopol^®^-based topical gels [51]. The comparable rheological profiles indicate that incorporation of imiquimod-loaded transferosomes did not substantially alter the flow behavior of the gel vehicle. Similarly, the maximum adhesive force was comparable between IQ-TF and Blank-TF (216 ± 3.5 and 175 ± 3.9 mN, respectively), further suggesting that imiquimod incorporation did not substantially modify the mechanical properties of the gel. Overall, the preservation of the rheological and adhesive characteristics of the vehicle may favor adequate spreading and prolonged contact with the skin, supporting the suitability of IQ-TF for localized topical drug delivery [17].

An ideal topical formulation should overcome the stratum corneum barrier and preferentially promote drug accumulation within viable skin layers such as the dermis where *Leishmania* amastigotes reside, while minimizing transdermal permeation to avoid systemic toxicity [52,53]. In this context, skin permeation and tape-stripping studies were performed to determine whether the physicochemical characteristics of the developed transferosome system would promote imiquimod retention and permeation within the skin.

The permeation experiments demonstrated that IQ-TF promoted the continuous transport of imiquimod across porcine skin throughout the 6 h experimental period, reaching a cumulative permeated amount of 11.52 ± 9.05 μg, with a steady-state flux (J_ss_) of 0.86 ± 0.13 μg/cm^2^/h, a permeability coefficient of 5 × 10^−4^ cm^2^/h, and a lag time of 0.894 ± 0.005 h. The long lag time indicates that an interaction between the formulation and the skin barrier occurs following application.

Although the steady-state flux obtained in the present study cannot be directly compared with that reported by Fernández-García et al. [12] because of differences in drug physicochemical properties, formulation composition, skin model, and experimental conditions, both studies consistently demonstrate that transferosome systems promote sustained drug transport across biological skin while maintaining localized topical delivery rather than unrestricted transdermal permeation. Fernández-García et al. reported a higher steady-state flux for amphotericin B-loaded transferosomes across mouse skin. However, as demonstrated by the tape-stripping experiment, imiquimod was predominantly retained within the stratum corneum, while its distribution within deeper viable skin layers could not be directly determined.

The permeation profile observed is consistent with the characteristic transport mechanism of transferosomes. Transferosomes primarily penetrate the skin by deforming between the dead anucleate keratinocytes. Incorporating edge activators increases the fluidity of the transferosome phospholipid bilayer. This allows the vesicles to undergo reversible deformation and migrate through intercellular pathways that are considerably smaller than their hydrodynamic diameter. Meanwhile, phospholipids and surfactants can temporarily interact with the lipid domains of the stratum corneum, increasing lipid fluidity, facilitating drug diffusion, and preserving the skin’s overall barrier integrity [12,48,54].

This drug disposition profile is particularly relevant for cutaneous leishmaniasis because the therapeutic objective is to establish sufficient drug concentrations in the dermis where *Leishmania* amastigotes reside in macrophages, avoiding systemic absorption. Likewise, Fernández-García et al. [12] emphasized this concept, demonstrating that the success of transferosome-based formulations depends on their ability to localize the drug within cutaneous tissues rather than promoting extensive transdermal delivery. The tape-stripping analysis provided a layer-by-layer quantification of the imiquimod retained in the sequentially removed stratum corneum layers to assess the cutaneous distribution profile of IQ-TF [55,56]. However, this approach does not allow the distribution of the nanoformulation within deeper viable skin compartments to be directly determined. Therefore, whether IQ-TF reaches the deeper dermal and directly interacts with infected cells remains to be established. Future studies could consider imaging-based approaches, such as fluorescently labeled transferosomes combined with confocal microscopy and appropriate cellular markers, to further characterize the spatial localization of the nanoformulation within the skin.

Since in vitro activity alone is insufficient if therapeutic drug concentrations cannot be achieved in infected skin, the biological relevance of the skin delivery profile observed for IQ-TF is clear. IQ-TF resulted in increased antileishmanial activity both in vitro and in vivo. The in vitro experiments showed that IQ-TF demonstrated significantly higher antileishmanial activity than imiquimod against both promastigotes and intracellular amastigotes of *L*. (*L*.) *amazonensis*. This enhanced activity is reflected by the marked reduction in the EC_50_ values of IQ-TF compared to IQ in both parasite forms after 24 and 72 h. Against promastigotes, the EC_50_ decreased from 21,060 ± 1540 to 50 ± 2 ng/mL and from 12,560 ± 1410 to 4.55 ± 0.01 ng/mL after 24 and 72 h for IQ and IQ-TF, respectively. Similarly, against intracellular amastigotes, the EC_50_ decreased from 5800 ± 0.4 to 4 ± 2 ng/mL and from 30 ± 10 to 1.2 ± 0.7 ng/mL after 24 and 72 h, respectively.

However, this enhanced antileishmanial activity was accompanied by increased cytotoxicity toward host macrophages. Encapsulating imiquimod in transferosomes resulted in a markedly lower CC_50_ value (150 ± 1 ng/mL) compared with free imiquimod (35,490 ± 9540 ng/mL), indicating increased in vitro cytotoxicity. This effect may be related to the enhanced interaction of the transferosome system with cell membranes and the consequent increase in cellular exposure to imiquimod. The presence of sodium deoxycholate, used as an edge activator, may also contribute to the observed cytotoxicity due to its surfactant properties. Although this increased in vitro cytotoxicity represents a potential limitation of the formulation, it should be considered alongside the markedly enhanced antileishmanial activity of IQ-TF. Importantly, the selectivity indices varied according to parasite form and exposure time, indicating that the enhanced antileishmanial activity of IQ-TF was not uniformly accompanied by improved selectivity. In the topical in vivo study, no evident signs of local intolerance, such as skin irritation or erythema, or treatment-related body weight loss were observed under the experimental conditions used. These findings provide preliminary evidence of tolerability in the animal model, although further safety evaluation is warranted.

Therefore, the increased cytotoxicity of IQ-TF must be evaluated alongside its antileishmanial potency and selectivity indices. Although cell exposure increased, IQ-TF showed a significant decrease in EC_50_ values against both promastigotes and intracellular amastigotes. Consequently, the selectivity indices varied according to parasitic stage and incubation time rather than following a uniform trend relative to free imiquimod. Taken together, these findings suggest that, although the transferosome system enhances cellular interaction and antiparasitic efficacy, this advantage is tied to increased host–cell cytotoxicity [57].

Interestingly, Blank-TF exhibited intrinsic antileishmanial activity against promastigotes, with EC_50_ values of 100 ± 3 and 530 ± 30 ng/mL after 24 and 72 h, respectively. This finding is consistent with previous evidence showing that sodium deoxycholate can exhibit intrinsic antileishmanial activity among pharmaceutical excipients evaluated against *Leishmania* sp. [58]. Since sodium deoxycholate was incorporated into the transferosome formulation as an edge activator, it may contribute to the observed activity of Blank-TF. However, as Blank-TF contains both sodium deoxycholate and phospholipids, the present study does not allow us to determine the relative contribution of each individual component. Consequently, the enhanced antileishmanial activity observed for IQ-TF may reflect the combined contributions of imiquimod and the transferosome components, rather than improved drug delivery alone.

The encouraging in vitro findings were evaluated in a murine model of cutaneous leishmaniasis to determine if the enhanced biological activity observed under controlled cellular conditions would translate into a therapeutic benefit in vivo.

IQ-TF significantly reduced the parasite burden in infected animals compared to untreated animals and produced therapeutic responses comparable to those observed with deoxycholate amphotericin B, a reference drug currently used to treat leishmaniasis. Importantly, no statistically significant differences were detected between topical IQ-TF and intraperitoneal AmB with respect to parasite load. While these findings do not demonstrate therapeutic equivalence due to differences in administration route, dose, and formulation, they suggest that topical IQ-TF may achieve a level of parasite control comparable to this established antileishmanial therapy under the evaluated experimental conditions.

This observation is particularly noteworthy given the substantial differences between the two therapeutic strategies. AmB was administered intraperitoneally, while IQ-TF was applied topically directly to the lesion. Furthermore, the transferosome formulation contained only 1.64 mg/g of imiquimod, which is approximately 30 times lower than the concentration of the drug in the commercial IQ cream originally developed for other dermatological conditions [43]. This cream has been investigated as a repurposed therapy for cutaneous leishmaniasis [19,24,27,42] and was included in the present study as a point of comparison. Despite the markedly lower drug concentration, IQ-TF produced a reduction in parasite burden comparable to the commercial formulation and systemic AmB (standard treatment).

These findings are particularly relevant considering that the experimental model was established using *L*. (*L*.) *amazonensis*, one of the most challenging etiological agents of cutaneous leishmaniasis in the Americas. This species is associated with not only localized ulcerative cutaneous lesions, but also diffuse cutaneous leishmaniasis. This severe, anergic form is characterized by uncontrolled parasite replication, heavily parasitized macrophages, poor cell-mediated immune responses, and limited therapeutic success [59]. Therefore, the significant reduction in parasite burden and the accompanying histopathological changes observed following IQ-TF treatment highlight the therapeutic potential of this nanoplatform against infections caused by a parasite species that remains particularly difficult to treat.

Although the present study was not designed to establish dose equivalence, these findings suggest that transferosome encapsulation can substantially improve the efficiency of local imiquimod delivery. This allows for therapeutically relevant responses to be achieved using considerably lower drug concentrations, thus avoiding the need for systemic administration.

Histopathological analysis further reinforced the parasitological findings. The absence of a significant reduction in lesion size despite the marked decrease in parasite burden may reflect, at least in part, the contribution of host inflammatory responses to lesion dimensions. A persistent and intense inflammatory infiltrate was observed throughout the dermis. Although some features of this response were compatible with parasite control and tissue repair, its persistence may have contributed to the maintenance of lesion size despite reduced parasitism. Thus, lesion size may not directly reflect parasite burden in this experimental model, and resolution of inflammation may occur more gradually than parasite clearance. Compared with untreated and Blank-TF-treated animals, lesions treated with IQ-TF exhibited fewer heavily parasitized macrophages, together with a predominance of lymphocytes and moderately parasitized macrophages. This finding suggests a modified inflammatory profile associated with reduced parasite burden. Polymorphonuclear cells were also more prominent in the IQ- and IQ-TF-treated groups than in the untreated infected and Blank-TF groups. Although this observation was qualitative, previous studies have shown that TLR7 signaling contributes to neutrophil effector functions during *L*. *major* infection, including reactive oxygen species production and neutrophil extracellular trap formation, and that early topical activation of TLR7 with imiquimod can improve parasite and lesion control [60]. Given that imiquimod is a known TLR7 agonist, these observations raise the possibility that TLR7-dependent mechanisms may contribute to the immunomodulatory effects observed with IQ and IQ-TF. However, because the present study did not directly evaluate TLR7 expression or activation, a causal link between TLR7 signaling and increased polymorphonuclear cell infiltration cannot be established. While inflammatory infiltrates were still present, these histopathological changes are consistent with partial infection control rather than complete lesion resolution, as expected in chronic experimental cutaneous leishmaniasis. In contrast, untreated and Blank-TF-treated animals displayed diffuse inflammatory infiltrates dominated by heavily parasitized macrophages, indicating an absence of meaningful therapeutic response in the absence of the active compound.

Interestingly, AmB-treated lesions produced comparable reductions in parasite burden but exhibited larger necrotic areas containing abundant cellular and parasitic debris. In contrast, IQ-TF induced histopathological changes that more closely resembled those observed with the commercial imiquimod formulation. These observations suggest that transferosome-mediated delivery of imiquimod contributes to parasite control while modulating the local inflammatory response.

From a translational perspective, the potential of IQ-TF can be evaluated based on the Target Product Profile (TPP) for cutaneous leishmaniasis (CL) established by the Drugs for Neglected Diseases initiative (DNDi). DNDi prioritizes safe, effective, field-adapted, topical, or oral treatments with short regimens that do not require a cold chain. In this context, IQ-TF exhibits several attributes that align with these priorities. Its topical application allows for the localized treatment of cutaneous lesions, potentially reducing the risk of systemic adverse effects compared to systemic therapies [61]. Furthermore, the 10-day treatment regimen evaluated here falls well within the ideal duration proposed by the DNDi TPP for topical therapies (≤14 days). This offers a practical advantage over conventional approaches, which often require prolonged treatment courses, frequent healthcare visits, or parenteral administration.

However, the alignment of IQ-TF with the TPP is preliminary. As the present study evaluated a single parasite species in an experimental murine model, its results do not apply to the diverse *Leishmania* species or patient populations covered by the TPP. Similarly, while the formulation was well tolerated under our experimental conditions, its long-term stability under tropical conditions, including independence from a cold chain, as well as its affordability, scalability, and field feasibility, remain to be demonstrated. Thus, further studies are required to determine its true potential for translation into a field-adapted therapy for CL.

Overall, the in vitro and in vivo findings are consistent with the improved pharmaceutical and skin delivery properties observed throughout this study. The enhanced antileishmanial activity observed against both promastigotes and intracellular amastigotes, the significant reduction in parasite burden, and the favorable histopathological changes identified in vivo suggest that delivery of imiquimod using transferosomes improves its therapeutic performance by increasing its local availability at the site of infection. Notably, these biological effects were achieved using a topical formulation containing approximately 30-fold less imiquimod than the commercial 5% cream, producing therapeutic responses comparable to those obtained with systemic deoxycholate AmB. Although additional mechanistic and pharmacodynamic studies are needed to elucidate the involved pathways, the present findings suggest the use of IQ-loaded transferosomes as a promising topical nanoplatform for treating cutaneous leishmaniasis.

## Figures and Tables

**Table 1:** Physicochemical characterization of IQ-loaded and blank transferosomes.

Samples	Particle Size (nm)	Zeta Potential (mV)	PDI	EE (%)
**IQ-TF**	596 ± 73	−15.2 ± 1.8	0.2 ± 0.02	71.2 ± 18.7
**Blank-TF**	574 ± 9	−23.6 ± 0.8 *	1.0 ± 0.5 *	-

Abbreviations: nm: nanometers; mV: millivolts; PDI: polydispersity index; IQ-TF: IQ-loaded transferosome; EE: encapsulation efficiency; Blank-TF: blank transferosome. * Unpaired Student t-test *p* < 0.05—Blank-TF vs. IQ-TF.

**Table 2:** Permeation parameters of IQ-loaded transferosomes (IQ-TFs) across excised porcine skin determined from the in vitro permeation study. Results are presented as the mean ± standard error of three independent experiments.

Parameter	IQ-TF Gel
**J_SS_ (** **μ** **g/cm^2^/h)**	0.858 ± 0.132
**Lag time (h)**	0.894 ± 0.005
**Permeability coefficient (cm^2^/h)**	5 × 10^−4^ ± 1 × 10^−4^
**Diffusion coefficient (cm^2^/h)**	6.8 × 10^−4^ ± 0.1 × 10^−4^

**J_SS_:** Steady-state flux.

**Table 3:** EC_50_ and CC_50_ caused by IQ-TF, Blank-TF, IQ, and AmB against *L.* (*L*.) *amazonensis* promastigote, amastigote forms and macrophages at 24 and 72 h of incubation. Results are shown as the mean ± standard error from three independent experiments.

Samples	EC_50_ (ng/mL)				CC_50_ (ng/mL)	
	*L*. (*L*.) *amazonensis*				Macrophages	
	Promastigote		Amastigote			
	24 h	72 h	24 h	72 h	24 h	72 h
**IQ-TF** **SI**	50 ± 2 **3**	4.55 ± 0.01 **59.34**	4 ± 2 **37.5**	1.2 ± 0.7 **225**	150 ± 1	270 ± 110
**Blank-TF** **SI**	100 ± 3 **11.9**	530 ± 30 **1.1**	N/A **-**	N/A **-**	1190 ± 30	570 ± 40
**IQ** **SI**	21,060 ± 1540 **1.69**	12,560 ± 1410 **1.43**	5800 ± 0.4 **6.12**	30 ± 10 **600.0**	35,490 ± 9540	18,000 ± 5010
**AmB** **SI**	40 ± 1 **74.5**	56.3 ± 0.3 **25.04**	3 ± 2 **993.3**	10 ± 2 **141**	2980 ± 70	1410 ± 90

EC_50_: Effective Concentration 50%; CC_50_: Cytotoxic Concentration 50%; IQ-TF: IQ-loaded transferosome; Blank-TF: blank transferosome; IQ: imiquimod; AmB: amphotericin B; SI: Selectivity Index.

## Data Availability

The data presented in this study are available on request from the corresponding author. The data are not publicly available due to privacy.
